# Site suitability analysis for potential agricultural land with spatial fuzzy multi-criteria decision analysis in regional scale under semi-arid terrestrial ecosystem

**DOI:** 10.1038/s41598-020-79105-4

**Published:** 2020-12-16

**Authors:** Barış Özkan, Orhan Dengiz, İnci Demirağ Turan

**Affiliations:** 1 Department of Industrial Engineering, Faculty of Engineering, Ondokuz Mayıs University, 55139 Samsun, Turkey; 2 Department of Soil Science and Plant Nutrition, Faculty of Agriculture, Ondokuz Mayıs University, 55139 Samsun, Turkey; 3 Department of Geography, Faculty of Economic, Administrative and Social Sciences, Samsun University, 55139 Samsun, Turkey

**Keywords:** Environmental sciences, Solid Earth sciences

## Abstract

The main purpose of this study is to identify suitable potential areas for agricultural activities in the semi-arid terrestrial ecosystem in the Central Anatolia Region. MCDA was performed in fuzzy environment integrated with GIS techniques and different geostatistical interpolation models, which was chosen as the basis for the present study. A total of nine criteria were used, as four terrain properties and five soil features to identify potential sites suitable for agriculture lands in Central Anatolia which covers approximately 195,012.7 km^2^. In order to assign weighting value for each criterion, FAHP approach was used to make sufficiently sensitive levels of importance of the criteria. DEM with 10 m pixel resolution used to determine the height and slope characteristics, digital geology and soil maps, CORINE land use/land cover, long-term meteorological data, and 4517 soil samples taken from the study area were used. It was identified that approximately 30.7% of the total area (59,921.8 ha) is very suitable and suitable for potential agriculture activities on S1 and S2 levels, 42.7% of the area is not suitable for agricultural uses, and only 27% of the area is marginally suitable for agricultural activities. Besides, it was identified that 34.8% of the area is slightly suitable.

## Introduction

Lands are one of the most important wealth of countries. Their quality and quantity are directly related to agricultural development and food safety. Therefore, sustainable agricultural production is the most important goal of developed or developing countries' agricultural policies. On the other hand, the pressure on the lands is increasing day by day with the increasing population. Particularly, the socio-economic needs of the rapidly growing population in developing countries forced the allocation of land resources for different uses for food production as the main goal. Therefore, the basis of the socio-economic development of countries depends on the abundance of natural resources and policies of using these resources. In addition, the pressure of growing population and competition arising from differences in land use requires more efficient land use and management. Rational and sustainable land use is an important issue for the benefit of the present and future population for land users and decision makers interested in the conservation of land resources. However, especially fertile farmlands have been negatively affected under the influence of industrialization, urbanization and wrong land use. Also, these fertile farmlands are exposed to excessive fertilization, disinfestation, domestic and industrial wastes. This pressure on soils leads to irreversible consequences.

Amount of arable land in the Turkey was about 27.5 million ha in the ‘80 s. According to the data of recent years, approximately 31% of the total land area of 78 million hectares in Turkey, i.e., approximately 24 million ha is considered as agricultural land^1^. Turkey has been faced the risk of land degradation and inappropriate use due to natural (i.e. climatic, topographic) and anthropogenic conditions. Especially the loss of agricultural land by erosion causes irreversible consequences. This case has been particularly felt in the Central Anatolia Region. This is why, identification of climate, vegetation, soil, and topographic features to make the most suitable decisions on land use and identification of suitable areas for cultivation land to reveal the correct uses by making comparisons between different lands, land evaluation and land use planning studies are quite important.

Identifying the suitability and quality of the lands has great importance for deciding on the use of land according to its potential and protecting natural resources for future generations. In this case, especially potential agricultural land should be identified and land use planning should be performed to make rational analysis and evaluation of fast, accurate, sufficient information and data about soil and land resources by using today's technologies^2^.

The development of methodologies facilitating the quantification of land suitability has been the main objective of the studies assessing the land evaluation^3–5^. In general, land evaluation methods are divided into two as qualitative methods based on expert knowledge and quantitative models based on simulation models^6^. Quantitative models are highly detailed for land performance and they often require much data, time and cost. On the other hand, land and soil features in the identification of agricultural land suitability in qualitative approaches are expressed in mathematical formulas. In this context, the evaluation of land suitability for agricultural activities is naturally regarded as a complex problem with multiple criteria. In other words, an evaluation approach involving multiple criteria would be more appropriate for land evaluation analysis studies. Today, besides the current techniques such as remote sensing and Geographic Information Systems (GIS), these challenges can be overcome by using approaches such as Multi-Criteria Decision Analysis (MCDA) to make rational analyses and evaluations^7–12^. Analytic Hierarchy Process^13^ (AHP) assigning weights to evaluation criteria belongs to often used MCDA methods. AHP is capable of identifying and incorporating inconsistencies in decision-making^14^. Typically, a priority vector is calculated based on the pair-wise comparison rising from a value determined by experts on a 1–9 scale. On the other hand, setting the explicit numerical values to evaluation criteria may be difficult or imprecise^15^ in reality. As a result, the evaluation criteria usually cannot be assigned precisely and decision makers indicate their weights in linguistic terms^16^. Applying fuzzy logic accommodates a mathematical strength to cover the uncertainties related to human cognitive process^17^. Buckley (1985)^18^ integrates fuzzy sets with AHP for uncertainty contemplation. Moreover, this approach has been used for different problems, including prioritization of dimensions of visual merchandising^19^, assessment of mine security risk^20^, assessment of surface water quality^21^, evaluation of occupational stress^22^, location selection of chromite processing plant^23^, selection of optimum maintenance strategy^24^, and ranking of geological risks^25^.

Identification of potential land suitability classes in the MCDA approach is usually developed using a four-stage process. These stages are (i) indicator selection, (ii) indicator categorization and scoring, (iii) weighting the indicators according to their significance, and (iv) calculating the scores according to a selected model^5,12,26,27^. However, the developed land quality indexes generally applicable under certain purposes and environmental conditions on a limited scale^28^. Therefore, no land quality index can be used universally and suitable for all kinds of geography^29^. Hence, indices that can identify the land quality of land and soil properties, usage type and plant species for all geographies cannot be expected ^30,31^. Also, the development of a model that can represent all ecological variables and socio-cultural habits is not practically possible and in theory, it is not economical in terms of time, labour and cost^32^.

In this study, it is aimed to identify the potential areas suitable for agricultural activities by taking into the MCDA approach in the Fuzzy Analytic Hierarchy Process (FAHP) environment in Central Anatolia Region that has a semi-arid terrestrial ecosystem. In addition, the fundamental basis hypothesis of this study is not only assisting to identify lands suitable for agricultural applications but also it is aimed to assist sustainable use and management of lands by taking into consideration the characteristics of soils, which are the most important sensitive elements of arid and semiarid terrestrial ecosystem against to land degradation and desertification.

## Materials and methods

### Field description of the study area

**Figure 1:**
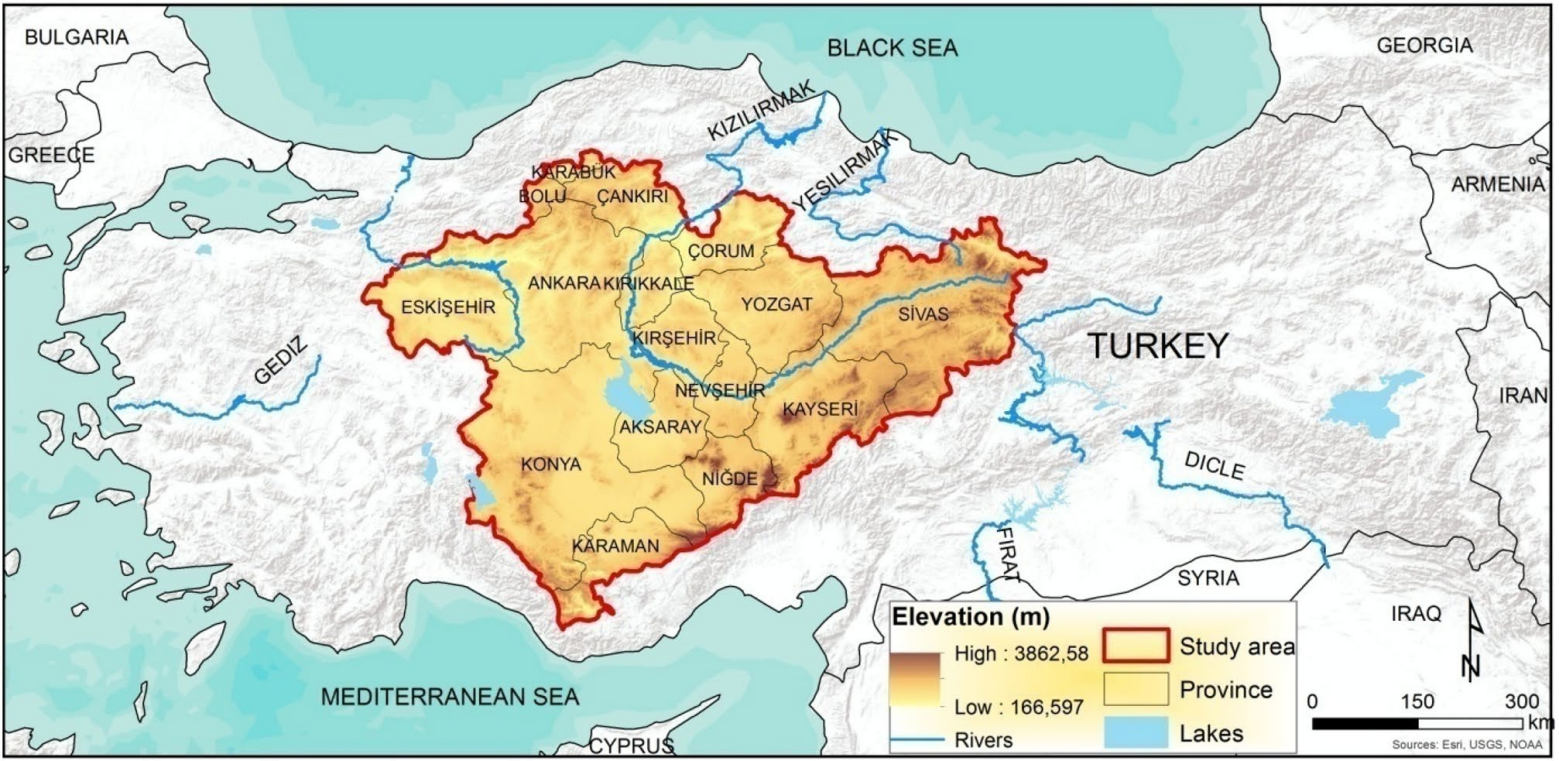
Location map of the study area (the map was created by the authors using the ArcGIS 10.2, http://esri.com).

The study area is between 30° 01′ 07″ and 38° 43′ 19″ east longitudes and 36° 18′ 08″ and 41° 07′ 11″ north latitudes and has a surface area of approximately 195,013 km^2^ (Fig. 1). The study area is within the borders of Bolu, Karabük, Çankırı, Çorum, Kırıkkale, Ankara, Eskişehir, Yozgat, Kırşehir, Aksaray, Konya, Niğde, Kayseri, Sivas, and Karaman provinces. The elevation of the study area is between 1600 and 3800 m above sea level. The region has an average elevation of 1200 m.

**Figure 2:**
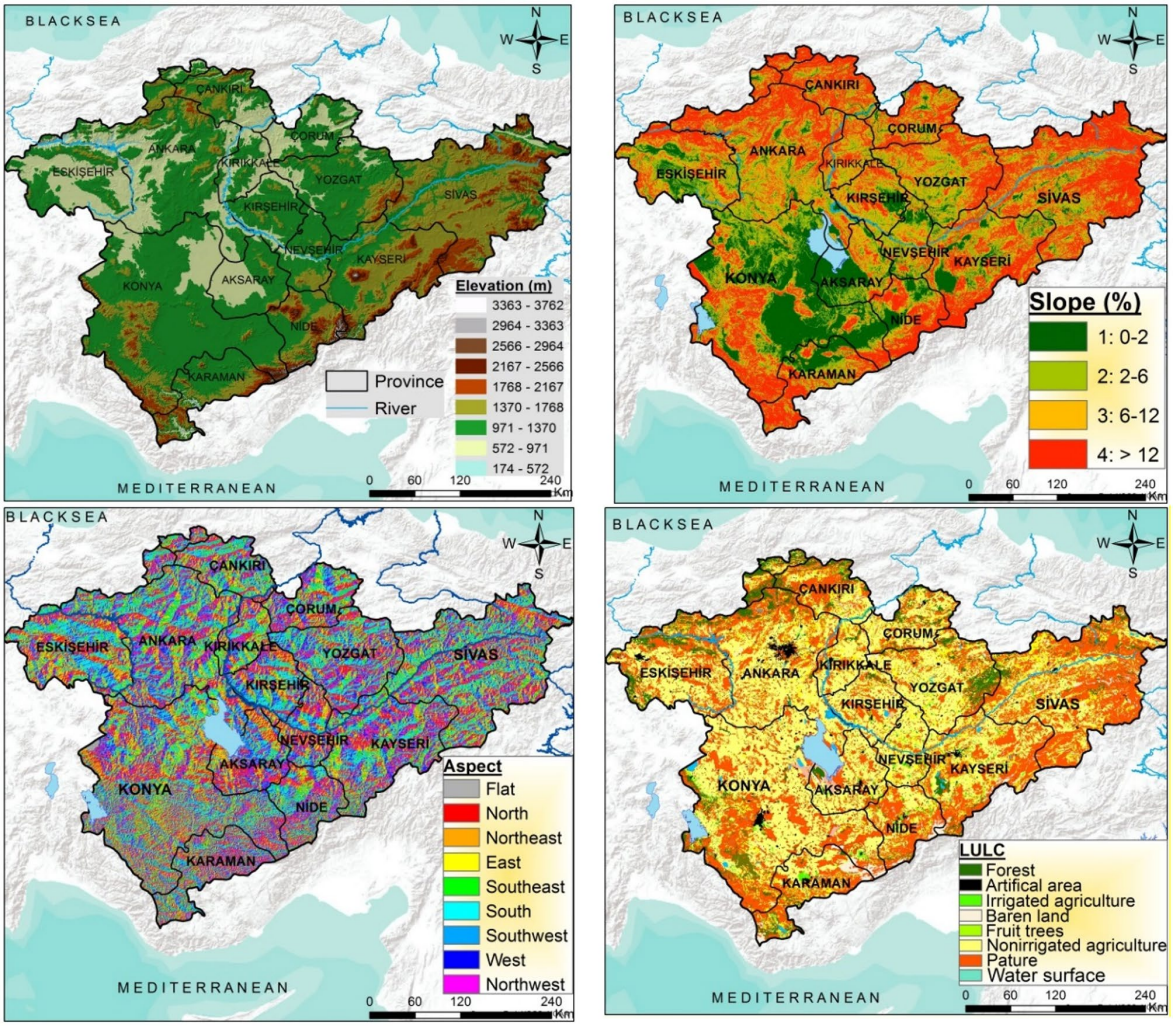
Elevation, slope, aspect and land cover-land use maps of the Central Anatolia Region (the maps were created by the authors using the ArcGIS 10.2, http://esri.com).

With an average slope of 9.6%, Central Anatolia Region is generally composed of flat fields and volcanic mountains rising in these fields. The area around Lake Tuz and Konya province have large flat fields. However, slope exceeds 30% towards the southeast and north of the study area, which is regarded as very steep. Also, 36.4% of the Central Anatolia Region is distributed in the southeast, south, and southwest aspect while, 38.1% is located in the north, northeast and northwest (Fig. 2).

The geological material of the Central Anatolia Region is composed of metamorphic, granitic and ophiolitic units, and this is regarded as Central Anatolian Crystalline Complex. The sedimentary origin main rock units of the Central Anatolian Crystalline Complex composed of Precambrian and early Paleozoic meta-clastic and meta-magmatic rocks (para-orthogneiss and rare carbonate arabantic schists) at the bottom, and late Paleozoic and Mesozoic meta-clastic rocks, calc-schist and marbles at the top. Non-metamorphic Upper-Maastrichtian-Lower Paleocene cover units on top of these units are covered by Paleocene-Eocene volcanic, volcaniclastic and carbonate rocks, Oligocene–Miocene evaporites, continental clastics with volcaniclastic and volcanic rocks represent the younger cover units of the Central Anatolian Crystalline Complex^33^.

The humid air of the seas cannot easily penetrate the Central Anatolia Region due to it is surrounded by high mountains. Therefore, the region is dominated by terrestrial climatic conditions where summers are usually hot and dry in summers and winters are cold and snowy. The degree of terrestrial climate increases towards the east with increasing altitude in the region. In this context, it can be said that semi-continental and semi-arid climatic conditions are more effective in the Central Anatolia Region. While the average annual temperature ranges from 8 °C to 12 °C at 0–1500 m elevations, it is well-known that the average temperature falls below 4 °C in higher elevations, such as Mount Erciyes. Central Anatolia Region is the region with the least rainfall in Turkey (Konya 326 mm, Karapınar 250 mm, Kayseri 375 mm, Kırşehir 378 mm, Çankırı 400 mm). The most rainfall occurs during the spring season in the east of the region and during the winter season in the west of the region. It can be said that semi-arid climatic conditions dominate most of the region when rainfall efficiency is considered. While the average annual relative humidity in the central part of Central Anatolia is around 55–60%, there are areas where relative humidity rises to 60–65% due to an increase in elevation and a decrease in air temperature. The relative humidity, which is reaching up to 80% in the winter season, is around 40–50% in summer. In addition to this, in summer some days, especially in August, the relative humidity in the air decreases up to 2%, which increases the evaporation extremely^34^.

The low biomass in grass (steppe) vegetation due to drought and terrestrial conditions that dominate the summer seasons in the lower elevations of the Central Anatolia Region caused the soil to be poor in organic substances. Sparse and arid forests are available, where oaks dominate the bottom part and black pines dominate the top part, in areas up to 2000 m starting over the steppes in Central Anatolia Region. However, anthropogenic steppes have become dominant since most of these forests have been destroyed^35^. According to ecological conditions in Central Anatolia, steppe fields are available in the area starting from Konya-Eregli plains in the south and extending to the Eskişehir Plain along the Sakarya and Porsuk brooks from the northwest of Lake Tuz^36^. The dominant species that make up the steppe vegetation in Central Anatolia are; *Artemisia fragrans, Thymus squarrosus, Festuca valesiaca, Ambyliopyrum muticum, Agropyron divaricatum, Hordeum murinum, Onopordon acanthium, Satureja cuneifolia, Stipa *sp.*, Bromus *sp.,* Festuca *sp.,* Alyssum *sp.,* Ajuga* sp.,* Centaurea *sp.*, Galium *sp.*, Medicago *sp.,* Marrubium *sp.*, Nigella *sp.*, Papaver *sp.,* Convolvulus *sp.*, Crucianella *sp.,* Trifolium* sp.*, Salvia *sp.,* Senecio *sp.*, Sideritis *sp.,* Ziziphora *sp.,* Leontodon asperrimum’dur. Başlıca çalılar ise; Prunus spinosa, Jasminum fruticans, Rosa sulphurea, Crataegus orientalis, Lonicera etrusca, and Clematis vitalba*^36^. According to the Central Anatolia Region CORINE-2012 land use-land cover classification, 40% of the area is agricultural area and this is followed by pasture area with 35.7% and forests with %14.5 (Fig. 2).

### Soil sampling and soil physico-chemical analyses

**Figure 3:**
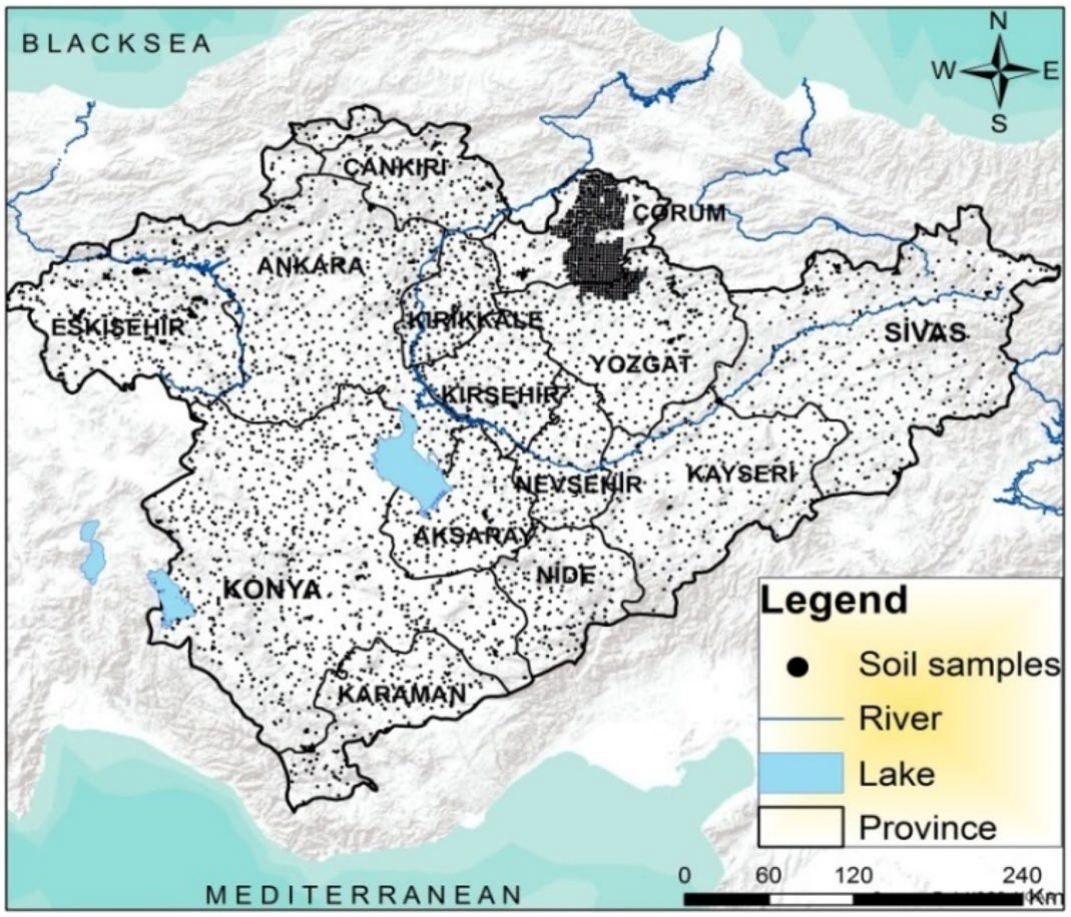
Soil samples in the study area (the map was created by the authors using the ArcGIS 10.2, http://esri.com).

Total 4517 coordinated soil samples were taken from a depth of 0–30 cm from the study area (Fig. 3). Samples brought to the laboratory are prepared for physical and chemical analysis after the separation from roots and coarse particles. Soil properties were determined with the following methods: soil particle size distribution by the hydrometer method; pH and electrical conductivity (EC) in 1:2.5 (w/v) in soil/water suspension by pH-meter and EC-meter, respectively; CaCO_3_ content by the volumetric method^37^. All soil samples were sieved through a 150 μm mesh before determination of the total organic matter content with the wet oxidation (Walkley-Black) method with K_2_Cr_2_O_7_^38^.

### Interpolation analyses and descriptive statistics

In this study, different interpolation methods (Inverse Distance Weighing-IDW with the weights of 1, 2, 3 and radial basis function-RBF with thin plate spline (TPS), simple kriging (OK) with spherical, exponential and gaussian variograms, ordinary kriging (OK) with spherical, exponential and gaussian variograms, universal kriging (OK) with spherical, exponential and gaussian variograms) were applied for predicting the spatial distribution of soil some parameters texture, pH, bulk density, lime and organic matter content) with ArcGIS 10.2.2v.

In the present study, root mean square error (RMSE) was used to assess and figure out the most suitable interpolation model. That’s why, the lowest RMSE indicates the most accurate prediction. Estimates are determined by using Eq. (1).

1 $$\mathrm{RMSE}=\sqrt{\frac{\sum {({\mathrm{z}}_{{\mathrm{i}}^{*}}-{\mathrm{z}}_{\mathrm{i}})}^{2}}{\mathrm{n}}}$$

where; RMSE: root mean square error, Zi is the predicted value, Zi* is the observed value, and n is the number of observations.

Descriptive statistics as minimum, maximum, mean, standard deviation, skewness, kurtosis coefficient and coefficients of variation of physico-chemical properties of surface soil samples were calculated.

### Multi criteria assessment approach

The selection of indicators to be used to identify suitable areas for potential agricultural lands is very important^29^. Because there are many properties that affect the quality of the lands in different agricultural uses in varying proportions and it is not possible to use all of them^39^. Regarding this issue, Doran and Parkin (1996)^32^ proposed to use as few parameters as possible in modeling approaches. As a matter of fact, it is known that there is a high correlation between some physical, chemical and biological properties. As using all of them at the same time as a criterion is practically impossible, it is also known that it is contrary to the basic principles of the land evaluation measurement paradigm^26^. This is why, taking into account the indicator eligibility of representing one or more of the soil characteristics, nine different evaluation parameters, which are affecting plant growth in agricultural suitability index and also proposed/used by De La Rosa et al. (1981)^40^, Dengiz (2007)^41^, Hazelton and Murphy (2007)^42^, Iojă et al. (2014)^43^, Zhang et al. (2015)^29^, Mustafa et al. (2017)^44^, Demirağ Turan and Dengiz (2017)^45^, Aldababseh et al. (2018)^46^, were selected. In this current study, the following soil characteristic: soil texture, OM, BD, pH, and CaCO_3_ were suggested by many researchers due to their effects on water holding capacity, pore size, soil structure, and aggregate stability, root growth, soil fertility, availability plant nutrient elements, etc^47–49^. Demirağ Turan et al. (2019)^50^ indicated that soil organic matter content represents a key indicator for soil quality, both for agricultural functions (i.e. production and economy) and for environmental functions (i.e. carbon sequestration and air quality). Lime content of soil in cultivated area is main reason for available nutrient element behaviour and influences also soil reaction. According to Eyüpoğlu (1999)^51^ lime (CaCO_3_) content of Turkey's territory has been studied and it was determined that 58.6% of the territory is calcareous soils due to parent material and low precipitation and located mostly around Central Anatolia Region. In addition, some land characteristics such as slope, depth, erosion, parent material have also crucial role for arable lands. Particularly increasing of slope degree negatively influences the drainage-irrigation and field traffic or mechanization practices^41,52^. In addition, the high slope degree causes along the risk of soil erosion and this leads to organic matter and nutrient loss, especially in surface soil^53^. For these reasons, slope factor has been adopted and used as a limiting factor for land suitability in the arable land according to the FAO Framework^54^. Selected parameters and activities for identifying potential agricultural areas are presented in Table 1.

**Table 1:** Selected criteria for land suitability for agricultural usage and their effectiveness.

	Criterion	Effectiveness	Literatures
Land criteria	Depth	Root development, water retention	Sarkar et al., 2014^55^; Bandyopadhyay et al. (2009)^56^
	Slope	Field traffic, runoff,	FAO, 1976^57^; Feizizadeh and Blaschke, 2012^58^
	Erosion	Soil loss	Dengiz, 2007^41^; Demirağ Turan and Dengiz, 2017^45^
	Parent material	Soil formation	Bera et al., 2017^59^; Pramanik, 2016^60^; Dengiz et al., 2019^61^
Soil criteria	Texture	Infiltration, structure development, soil–water relationship	Ahmed et al., 2016^4^; Ashraf et al., 2002^62^
	Bulk density	Soil compaction, aeration, infiltration	Şeker ve Işıldar, 2000^63^; Pagliai et al., 2003^64^
	Lime content	Availability of nutrient elements, pH regulation in acid condition	Gezgin and Hamurcu 2006^65^; Feizizadeh and Blaschke, 2012^58^
	pH	Availability of nutrient elements, microbial activity	Baridón et al., 2014^66^; Feizizadeh and Blaschke, 2012^58^
	Organic Material	Soil quality, biological activity	Riley et al., 2008^67^; Kurzatkowski, 2004^68^; Bandyopadhyay et al., 2009^56^; Guo et al., 2015^69^

Karaca et al. (2020)^70^ reported that it was generally accepted that land and soil quality indicators can be separated as either inherent or dynamic. The inherent factors are for example soil texture or mineralogical composition, while the dynamic characteristic pointed out that dynamic factors are considered to evaluate how soil management decisions affect soil properties. This study was performed at reginal scale. That is why, mostly the inherent indictors were preferred for site suitability of potential agricultural land. A total of nine criteria were used, as four terrain properties including slope, depth, erosion, and parent material and five soil features including organic matter, bulk density (BD), texture, pH and lime content (CaCO_3_), in this study to identify potential sites suitable for agriculture uses. Also, classifications for each criterion have been created and scores between 1 and 4 are given for classifications each of these classifications to identify the suitability for potential agricultural activities. If criteria classifications are at an optimum level of suitability for potential agriculture uses, they are scored as 1 and if they have low suitability, they are scored as 4. Between two values is evaluated as classifying factor and degree (Table 2).

**Table 2:** Criteria, sub-criteria and their index values used for land suitability classes of agriculture usages.

Land criteria									
C. Parent material		C2. Erosion (ton/ha/year)		C3. Soil depth (cm)		C4. Slope (%)			
Class	Value	Class	Value	Class	Value	Class	Value		
Alluvial deposits	1	0–5	1	0–20	4	0–2	1		
Basic-ultrabasic magmatic and eruptions, melange, ophiolitic and serpentine, shale, metamorphic rocks such as schist, phyllite clay stone, marl	2	5–10	2	20–50	3	2–6	2		
Siltstone, mudstone, conglomerate, travertine, limestone, dolomite, marble	3	10–20	3	50–90	2	6–12	3		
Acid magmatic, cherty, gneiss, dunes, volcanic ashes, tuff, agglomerate, breccia, evaporates, pebble stone, sand stone	4	> 20	4	> 90	1	> 12	4		

Soil criteria									
C5. Organic matter (%)		C6. Bulk density (g/cm^3^)		C7. Texture		C8. pH		C9. CaCO_3_ (%)	
Class	Value	Class	Value	Class	Value	Class	Value	Class	Value
> 3	1	1.0–1.2	1	Medium (L, Si, SiL, fSL)	1	6.5–7.5	1	0–5	1
3–2	2	1.2–1.4	2	Fine (C < %45, CL, SiL, SCL)	2	5.5–6.5	2	5–10	2
2–1	3	1.4–1.55	3	Very fine (fC > %45, SiCL, SC)	3	7.5–8.2	3	10–20	3
0–1	4	> 1.55	4	Coarse (S, SL, LS)	4	< 5.5- > 8.2	4	> 20	4

The relative importance of these criteria should be determined (weighting) since they are not equally effective in identifying potential agriculture areas. In this study, the FAHP method was used to weight the criteria. Detailed information about the method is included in the following sections of the study.

Once the relative importance levels of the criteria have been determined, the Weighted Linear Combination (WLC) method was used for the identification of potential agricultural areas. WLC is also known as simple additive weighting (SAW), weighted calculation, weighted linear mean and weighted thrust^71^. WLC method calculates the value of the suitability of a potential region by using the formula in Eq. (2).

2 $${\mathrm{S}}_{\mathrm{i}}=\sum_{\mathrm{k}=1}^{\mathrm{l}}{\mathrm{w}}_{\mathrm{k}}{\mathrm{a}}_{\mathrm{ik}}$$

where (Eq. 2), ${\mathrm{S}}_{\mathrm{i}}$ represents the suitability value of the potential agricultural area; ${\mathrm{w}}_{\mathrm{k}}$ represents the relative importance of the criterion k, ${\mathrm{a}}_{\mathrm{ik}}$ represents the standard value under criterion k in i suitability area and l represents the total number of criteria^72^.

After studies carried out considering the frequency distribution of values ​​and statistical information, it is considered appropriate to be shown in 5 classifications with Natural Breaks Jenks method^73^. This method is used when data is not evenly distributed, there are huge differences between values and differences between classifications that need to be presented explicitly. Suitability classifications and index values ​​for these classifications for potential agricultural areas are shown in Table 3.

**Table 3:** Land suitability classes and their index values for agriculture usages.

Class	Index	Definition
S1	1.33–1.91	Very high suitable
S2	1.92–2.24	High suitable
S3	2.25–2.52	Marginally suitable
N1	2.53–2,79	Currently non suitable
N2	2.80–3.95	Permanently non suitable

### Fuzzy logic sets

Zadeh (1965)^74^ first introduced the fuzzy set theory, whose application enables decision makers to effectively deal with the uncertainties. In classical set theory, an element either belongs or does not belong to the set. Fuzzy sets are sets whose elements have degrees of membership. A triangular fuzzy number (TFN) is a type of fuzzy number and, according to Van Laarhoven and Pedrycz (1983)^75^, should possess the some basic properties. The membership function of the TFN is as follows^76^:

A fuzzy number $\stackrel{\sim }{A}$ on ${\mathbb{R}}$ to be TFN if it is membership function $x\in \stackrel{\sim }{A},{\mu }_{\stackrel{\sim }{A}} \left(x\right):{\mathbb{R}}\to \left[\mathrm{0,1}\right]$ is equal to

3 $${\mu }_{\stackrel{\sim }{A}} (x)=\left\{\begin{array}{l}(x-l)/(m-l), l\le x\le m,\\ (u-x)/(u-m), m\le x\le u,\\ 0, otherwise\end{array}\right.$$

where $l$ and $u$ stand fort he lower and upper bounds of the fuzzy number $\stackrel{\sim }{A}$, respectively, and $m$ for the modal value. The TFN can be denoted by $\stackrel{\sim }{A}=(l,m,u)$ and the following is the operational laws of two TFNs ${\stackrel{\sim }{A}}_{1}=\left({l}_{1},{m}_{1}, {u}_{1}\right)$ and ${\stackrel{\sim }{A}}_{2}=\left({l}_{2},{m}_{2}, {u}_{2}\right)$.

Addition of fuzzy number $\oplus$

4 $${\stackrel{\sim }{A}}_{1}\oplus {\stackrel{\sim }{A}}_{2}=({l}_{1}+{l}_{2}, {m}_{1}+{m}_{2},{u}_{1}+{u}_{2})$$

Multiplication of a fuzzy number $\otimes$

5 $${\stackrel{\sim }{A}}_{1} \otimes{\stackrel{\sim }{A}}_{2}=\left({l}_{1}{l}_{2}, {m}_{1}{m}_{2},{u}_{1}{u}_{2}\right),$$

for $${l}_{1}{l}_{2}>0; {m}_{1}{m}_{2}>0; {u}_{1}{u}_{2}>0$$

Subtraction of a fuzzy number $\ominus$

6 $${\stackrel{\sim }{A}}_{1}\ominus {\stackrel{\sim }{A}}_{2}=\left({l}_{1}-{u}_{2}, {m}_{1}-{m}_{2},{u}_{1}-{l}_{2}\right),$$

Division of a fuzzy number $\oslash$

7 $${\stackrel{\sim }{A}}_{1}\oslash {\stackrel{\sim }{A}}_{2}=({l}_{1} / {u}_{2}, {m}_{1} / {m}_{2},{u}_{1} / {l}_{2}), $$

for $${l}_{1}{l}_{2}>0; {m}_{1}{m}_{2}>0; {u}_{1}{u}_{2}>0$$

Reciprocal of a fuzzy number

8 $${{\stackrel{\sim }{A}}_{1}}^{-1}={\left({l}_{1},{m}_{1}, {u}_{1}\right)}^{-1}=(1 / {u}_{1}, 1 / {m}_{1},1 / {l}_{1})$$

for $${l}_{1}{l}_{2}>0; {m}_{1}{m}_{2}>0; {u}_{1}{u}_{2}>0$$

### Fuzzy-AHP

The AHP method developed by Thomas L. Saaty is a mathematical method considering the priorities of the group or individual, and evaluating qualitative and quantitative variables together in decision making^13^. The AHP method is frequently used in solving multiple criteria decision-making problems since it is easy to understand and includes simple mathematical calculations. However, the use of linguistic expressions such as "very good", "good", "bad", and "very bad" instead of using numbers while decision-makers making pairwise comparisons during the implementation of the AHP method, is easier and better reflects the thinking style. FAHP is introduced by integrating the traditional method with fuzzy logic in order to prevent this deficiency of AHP method in decision making.

There are different FAHP methods proposed by different researchers in the literature^18,75,77–79^. Buckley (1985)^18^ calculates the fuzzy weights of the criteria using the geometric mean method. In this present study, Buckley's method was used to calculate criterion weights. The steps of the method implemented using Buckley's method are as follows.

### Stage 1

Pairwise comparison matrices are created between all criteria in the hierarchical structure. Linguistic expressions corresponding to pairwise comparison matrices are assigned by asking which is more important for each of the two criteria as in matrix $\stackrel{\sim }{A}$.

9 $$\stackrel{\sim }{A}=\left[\begin{array}{cccc}1& {\stackrel{\sim }{a}}_{12}& \cdots & {\stackrel{\sim }{a}}_{1n}\\ {\stackrel{\sim }{a}}_{21}& 1& \cdots & {\stackrel{\sim }{a}}_{2n}\\ \vdots & \vdots & \ddots & \vdots \\ {\stackrel{\sim }{a}}_{n1}& {\stackrel{\sim }{a}}_{n2}& \cdots & 1\end{array}\right]=\left[\begin{array}{cccc}1& {\stackrel{\sim }{a}}_{12}& \cdots & {\stackrel{\sim }{a}}_{1n}\\ 1/{\stackrel{\sim }{a}}_{21}& 1& \cdots & {\stackrel{\sim }{a}}_{2n}\\ \vdots & \vdots & \ddots & \vdots \\ 1/{\stackrel{\sim }{a}}_{n1}& {\stackrel{\sim }{a}}_{n2}& \cdots & 1\end{array}\right]$$

where;

$$ \begin{gathered}   a_{{ij}}  = \left\{ \begin{gathered}   \tilde{1},\tilde{2},\tilde{3},\tilde{4},\tilde{5},\tilde{6},\tilde{7},\tilde{8},\tilde{9} \hfill \\   1 \hfill \\   \tilde{1}^{{ - 1}} ,\tilde{2}^{{ - 1}} ,\tilde{3}^{{ - 1}} ,\tilde{4}^{{ - 1}} ,\tilde{5}^{{ - 1}} ,\tilde{6}^{{ - 1}} ,\tilde{7}^{{ - 1}} ,\tilde{8}^{{ - 1}} ,\tilde{9}^{{ - 1}}  \hfill \\  \end{gathered}  \right. \hfill \\   criterion~i\,\, ~is\,\, ~relative\,\, ~importance\,\, ~to\,\, ~criterion~j \hfill \\   i = j \hfill \\   criterion~i~\,\, is\,\, ~relative\,\, ~less\,\, ~importance\,\, ~to\,\, ~criterion~j \hfill \\  \end{gathered}  $$

### Stage 2

Linguistic expressions in pairwise comparison matrices are converted to triangular fuzzy numbers. In this study, the scale created by Gumus (2009)^80^ was used for the conversion of linguistic expressions into triangular fuzzy numbers (Table 4).

**Table 4:** Triangular fuzzy conversion scale.

Fuzzy number	Linguistic scales	Scale of triangular fuzzy number	Scale of triangular reciprocal fuzzy number
$\stackrel{\sim }{1}$	Equal	(1,1,1)	(1,1,1)
$\stackrel{\sim }{2}$	Weak advantage	(1,2,3)	(1/3,1/2,1)
$\stackrel{\sim }{3}$	Not bad	(2,3,4)	(1/4,1/3,1/2)
$\stackrel{\sim }{4}$	Preferable	(3,4,5)	(1/5,1/4,1/3)
$\stackrel{\sim }{5}$	Good	(4,5,6)	(1/6,1/5,1/4)
$\stackrel{\sim }{6}$	Fairly good	(5,6,7)	(1/7,1/6,1/5)
$\stackrel{\sim }{7}$	Very good	(6,7,8)	(1/8,1/7,1/6)
$\stackrel{\sim }{8}$	Absolute	(7,8,9)	(1/9,1/8,1/7)
$\stackrel{\sim }{9}$	Perfect	(8,9,10)	(1/10,1/9,1/8)

### Stage 3

The geometric mean technique proposed by Buckley (1985)^18^ is used to calculate the fuzzy geometric mean and fuzzy weight of each criterion.

10 $${\stackrel{\sim }{r}}_{i}={\left({\stackrel{\sim }{a}}_{i1} \otimes {\stackrel{\sim }{a}}_{i2}\otimes\dots \otimes{ \stackrel{\sim }{a}}_{in}\right)}^{1/n}$$

11 $${\stackrel{\sim }{w}}_{i}={{\stackrel{\sim }{r}}_{i} \otimes \left({\stackrel{\sim }{r}}_{1} \oplus \dots \oplus { \stackrel{\sim }{r}}_{n}\right)}^{-1}$$

${\stackrel{\sim }{a}}_{in}$ is a fuzzy pairwise comparison value of criteria $i.$ and criteria $n$. Accordingly, ${\stackrel{\sim }{r}}_{i}$ is the geometric mean of the fuzzy pairwise comparison value of criteria $i.$ with each criterion. ${\stackrel{\sim }{w}}_{i}$ is fuzzy weight of criteria $i$. and expressed as ${\stackrel{\sim }{w}}_{i}=\left({lw}_{i},{mw}_{i},{uw}_{i}\right)$. ${lw}_{i}$, ${mw}_{i}$ and ${uw}_{i}$ represents the lower, middle and upper values of fuzzy weight of criteria $i,$ respectively.

### Stage 4

The criterion weights obtained are a triangular fuzzy number. Fuzzy numbers should be clarified to be converted into real numbers. The Center of Area (COA) method is one of the commonly used clarification methods because it is a simple and practical method^81^. The Best Non-fuzzy Performance (BNP) value of triangular fuzzy number ${\stackrel{\sim }{w}}_{i}$ is calculated by the formula in Eq. (12).

12 $${BNP}_{i}={lw}_{i}+\frac{\left({uw}_{i}-{lw}_{i}\right)+({mw}_{i}-{lw}_{i})}{3}, \forall i.$$

## Results and discussion

### Some soil physico-chemical properties

The some physical and chemical properties considered in this study showed variability as a result of dynamic interactions among natural environmental factors, including the degree of soil development and land use land cover types. Descriptive statistics of soil properties were given in Table 5. The value of pH in soil samples ranged between 6.40 and 9.47, BD had maximum 1.84, minimum 0.72 gr/cm^3^. In addition, minimum and maximum values of CaCO_3_ varied from 0.01% to 95.24% while, OM varied between 0.42% and 13.0%. Moreover, clay varied between 0.42% and 80.94%, silt varied between 1.01% and 81.16%, sandy varied between 1.38% and 93.84%. The texture components of basin soils exhibit normal distributions of clay, silt and sand content. On the other hand, other properties don’t exhibit normal distribution. The pH and BD show skewness characteristics to the left and OM, CaCO_3_, clay, silt and sand content have skewness characteristics to the right. The CV is the most important factor in defining the variability of soil properties^82^^.^ If the CV value is ≤ 15%, between 15 and 30%, or ≥ 30%, the variability is low, medium or high^83^. In this study, the lowest and highest CVs obtained for soil samples were 1.11% for BD, and 95.23% for CaCO_3_ content, respectively.

**Table 5:** Descriptive statistics some physico-chemical properties of soil samples.

Criteria	Mean	SD	CV	Variance	Min	Max	Skewness	Kurtosis
Clay (%)	29.03	13.43	80.51	190.45	0.43	80.94	0.36	− 0.22
Silt (%)	25.90	9.43	80.14	89.07	1.01	81.16	0.47	1.55
Sand (%)	43.98	16.68	92.46	278.51	1.38	93.84	0.24	− 0.26
BD (g/cm^3^)	1.37	0.13	1.11	0.02	0.72	1.84	-0.08	− 0.31
pH	7.60	0.48	5.03	0.23	6.40	9.47	-0.91	3.27
CaCO_3_ (%)	16.72	15.36	95.23	236.23	0.01	95.24	1.21	1.32
OM (%)	1.70	1.23	13.0	1.65	0.86	13.0	2.15	8.89

*OM* organic matter, *BD* bulk density, *SD* standard deviation, *CV* coefficient of variation, *Min* minimum, *Max* maximum.

### Determination of the suitable interpolation model

Fifteen interpolation models were applied in order to create soil criteria distribution maps and the lowest RMSE values found are given in Table 6. According to this, Kriging Simple Spherical model was determined as the most suitable for organic substance, Radial Basis Functions Spline with Tension was determined as the most suitable for volume weight, and Kriging Simple Gaussian model was determined as the most suitable for pH. Also, Radial Basis Functions Completely Regularized Spline was determined as the most suitable for lime and texture in order to generate their spatial distribution maps.

**Table 6:** Interpolation models and RMSE of soil criteria.

Interpolation models	Semivariogram modeller		Soil criteria				
			OM	BD	pH	CaCO_3_	Texture
Inverse distance weighting (IDW)	IDW-1		1.139	0.128	3.245	11.963	12.099
	IDW-2		1.197	0.133	3.559	12.226	12.465
	IDW-3		1.269	0.138	3.841	12.655	12.985
Radial basis functions (RBF)	TPS		6.285	1.955	4.837	11.798	12.098
	CRS		1.157	0.125	3.187	**11.745**	**12.012**
	SWT		1.172	**0.125**	3.183	11.787	12.013
Kriging	Ordinary	Gaussian	1.148	0.127	**3.096**	12.155	12.256
		Exponential	1.145	0.128	3.147	11.875	12.158
		Spherical	1.149	0.127	3.114	11.988	12.198
	Simple	Gaussian	1.132	0.363	3.136	12.231	19.897
		Exponential	1.135	0.135	3.793	11.910	12.688
		Spherical	**1.123**	0.139	3.222	12.094	13.280
	Universal	Gaussian	1.148	0.126	3.096	12.155	12.256
		Exponential	1.145	0.128	3.147	11.875	12.158
		Spherical	1.149	0.127	3.114	11.988	12.198

*TPS* thin plate spline, *CRS* completely regularized spline, *SWT* spline with tension, *OM* organic matter, *BD* bulk density.

### Determination of the criteria weight with FAHP

A decision-making team consisting of three experts was formed at application stage. Nine criteria were determined to be used in the study of identifying the areas suitable for potential agriculture in the study area with the literature support and the opinions of the expert team, and these criteria can be found in Table 2. These criteria representing the land and soil characteristics of the region have different importance levels in determining the areas suitable for agriculture. In this study, the FAHP method was used to weight the criteria.

Firstly, decision-makers were asked to make pairwise comparisons using the linguistic scale in Table 4 in determining the criterion weights. Values ​​obtained in the pairwise comparison process are determined as a result of the joint study of decision-making team members. The fuzzy numbers corresponding to the linguistic expressions obtained as a result of pairwise comparisons are given in Table 7. Pairwise comparison values ​​were converted to triangular fuzzy numbers using the scale in Table 8.

**Table 7:** Pairwise comparison matrix.

	C.1	C.2	C.3	C.4	C.5	C.6	C.7	C.8	C.9
C.1	1	${\stackrel{\sim }{3}}^{-1}$	${\stackrel{\sim }{3}}^{-1}$	${\stackrel{\sim }{5}}^{-1}$	${\stackrel{\sim }{5}}^{-1}$	${\stackrel{\sim }{2}}^{-1}$	${\stackrel{\sim }{3}}^{-1}$	${\stackrel{\sim }{2}}^{-1}$	$\stackrel{\sim }{2}$
C.2	$\stackrel{\sim }{3}$	1	${\stackrel{\sim }{3}}^{-1}$	${\stackrel{\sim }{3}}^{-1}$	$\stackrel{\sim }{3}$	$\stackrel{\sim }{3}$	$\stackrel{\sim }{3}$	$\stackrel{\sim }{5}$	$\stackrel{\sim }{5}$
C.3	$\stackrel{\sim }{3}$	$\stackrel{\sim }{3}$	1	${\stackrel{\sim }{1}}^{-1}$	$\stackrel{\sim }{3}$	$\stackrel{\sim }{5}$	$\stackrel{\sim }{3}$	$\stackrel{\sim }{3}$	$\stackrel{\sim }{5}$
C.4	$\stackrel{\sim }{5}$	$\stackrel{\sim }{3}$	1	1	$\stackrel{\sim }{5}$	$\stackrel{\sim }{5}$	$\stackrel{\sim }{2}$	$\stackrel{\sim }{5}$	$\stackrel{\sim }{5}$
C.5	$\stackrel{\sim }{5}$	${\stackrel{\sim }{3}}^{-1}$	${\stackrel{\sim }{3}}^{-1}$	${\stackrel{\sim }{5}}^{-1}$	1	$\stackrel{\sim }{3}$	${\stackrel{\sim }{3}}^{-1}$	$\stackrel{\sim }{3}$	$\stackrel{\sim }{5}$
C.6	$\stackrel{\sim }{2}$	${\stackrel{\sim }{3}}^{-1}$	${\stackrel{\sim }{5}}^{-1}$	${\stackrel{\sim }{5}}^{-1}$	${\stackrel{\sim }{3}}^{-1}$	1	${\stackrel{\sim }{3}}^{-1}$	$\stackrel{\sim }{3}$	$\stackrel{\sim }{5}$
C.7	$\stackrel{\sim }{3}$	${\stackrel{\sim }{3}}^{-1}$	${\stackrel{\sim }{3}}^{-1}$	${\stackrel{\sim }{2}}^{-1}$	$\stackrel{\sim }{3}$	$\stackrel{\sim }{3}$	1	$\stackrel{\sim }{2}$	$\stackrel{\sim }{5}$
C.8	$\stackrel{\sim }{2}$	${\stackrel{\sim }{5}}^{-1}$	${\stackrel{\sim }{3}}^{-1}$	${\stackrel{\sim }{5}}^{-1}$	${\stackrel{\sim }{3}}^{-1}$	${\stackrel{\sim }{3}}^{-1}$	${\stackrel{\sim }{2}}^{-1}$	1	$\stackrel{\sim }{3}$
C.9	${\stackrel{\sim }{2}}^{-1}$	${\stackrel{\sim }{5}}^{-1}$	${\stackrel{\sim }{5}}^{-1}$	${\stackrel{\sim }{5}}^{-1}$	${\stackrel{\sim }{5}}^{-1}$	${\stackrel{\sim }{5}}^{-1}$	${\stackrel{\sim }{5}}^{-1}$	${\stackrel{\sim }{3}}^{-1}$	1

**Table 8:** Fuzzy pairwise comparison matrix.

	C.1	C.2	C.3	C.4	C.5	C.6	C.7	C.8	C.9
C.1	(1, 1, 1)	(1/4, 1/3, 1/2)	(1/4, 1/3, 1/2)	(1/6, 1/5, 1/4)	(1/6, 1/5, 1/4)	(1/3, 1/2, 1)	(1/4, 1/3, 1/2)	(1/3, 1/2, 1)	(1, 2, 3)
C.2	(2, 3, 4)	(1, 1, 1)	(1/4, 1/3, 1/2)	(1/4, 1/3, 1/2)	(2, 3, 4)	(2, 3, 4)	(2, 3, 4)	(4, 5, 6)	(4, 5, 6)
C.3	(2, 3, 4)	(2, 3, 4)	(1, 1, 1)	(1, 1, 1)	(2, 3, 4)	(4, 5, 6)	(2, 3, 4)	(2, 3, 4)	(4, 5, 6)
C.4	(4, 5, 6)	(2, 3, 4)	(1, 1, 1)	(1, 1, 1)	(2, 3, 4)	(4, 5, 6)	(1, 2, 3)	(4, 5, 6)	(4, 5, 6)
C.5	(4, 5, 6)	(1/4, 1/3, 1/2)	(1/4, 1/3, 1/2)	(1/6, 1/5, 1/4)	(1, 1, 1)	(2, 3, 4)	(1/4, 1/3, 1/2)	(2, 3, 4)	(4, 5, 6)
C.6	(1, 2, 3)	(1/4, 1/3, 1/2)	(1/6, 1/5, 1/4)	(1/6, 1/5, 1/4)	(1/4, 1/3, 1/2)	(1, 1, 1)	(1/4, 1/3, 1/2)	(2, 3, 4)	(4, 5, 6)
C.7	(2, 3, 4)	(1/4, 1/3, 1/2)	(1/4, 1/3, 1/2)	(1/3, 1/2, 1)	(2, 3, 4)	(2, 3, 4)	(1, 1, 1)	(1, 2, 3)	(4, 5, 6)
C.8	(1, 2, 3)	(1/6, 1/5, 1/4)	(1/4, 1/3, 1/2)	(1/6, 1/5, 1/4)	(1/4, 1/3, 1/2)	(1/4, 1/3, 1/2)	(1/3, 1/2, 1)	(1, 1, 1)	(2, 3, 4)
C.9	(1/3, 1/2, 1)	(1/6, 1/5, 1/4)	(1/6, 1/5, 1/4)	(1/6, 1/5, 1/4)	(1/6, 1/5, 1/4)	(1/6, 1/5, 1/4)	(1/6, 1/5, 1/4)	(1/4, 1/3, 1/2)	(1,1,1)

After the pairwise comparison matrix was transformed into triangular fuzzy numbers, firstly ${\stackrel{\sim }{r}}_{i}$ value was calculated by using Eq. (10) to calculate the fuzzy weights of criteria. For ${\stackrel{\sim }{r}}_{1}$ as example:

$${\stackrel{\sim }{r}}_{1}={\left(1\times (\frac{1}{4}\right)\times\left (\frac{1}{4}\right)\times \left(\frac{1}{6}\right)\times\left (\frac{1}{6}\right)\times \left(\frac{1}{3}\right)\times\left (\frac{1}{4}\right)\times \left(\frac{1}{3})\times 1\right)}^{1/9}$$, $${(1\times (\frac{1}{3})\times (\frac{1}{3})\times (\frac{1}{5})\times (\frac{1}{5})\times (\frac{1}{2})\times (\frac{1}{3})\times (\frac{1}{2})\times 2)}^{1/9}$$,

$${(1\times (\frac{1}{2})\times (\frac{1}{2})\times (\frac{1}{4})\times (\frac{1}{4})\times 1\times (\frac{1}{2})\times 1\times 3)}^{1/9}=(\mathrm{0.331,0.449,0.659})$$

Similarly, the remaining ${\stackrel{\sim }{r}}_{i}$ values were calculated, there are:

$${\stackrel{\sim }{r}}_{2}=$$(1.361,1.825,2.364), $${\stackrel{\sim }{r}}_{3}=$$(2.000,2.633,3.217), $${\stackrel{\sim }{r}}_{4}=$$(2.333,2.984,3.566), $${\stackrel{\sim }{r}}_{5}=$$(0.819,1.058,1.379), $${\stackrel{\sim }{r}}_{6}=$$(0.533,0.708,0.938), $${\stackrel{\sim }{r}}_{7}=$$(0.956,1.351,1.876), $${\stackrel{\sim }{r}}_{8}=$$(0.404,0.548,0.769),$${\stackrel{\sim }{r}}_{9}=$$(0.230,0.280,0.367)

Then, ${\stackrel{\sim }{w}}_{i}$ values were calculated using Eq. (11). For ${\stackrel{\sim }{w}}_{1}$ as example:

$${\stackrel{\sim }{w}}_{1}=\left(\mathrm{0.331,0.449,0.659}\right)\otimes $$ $$(1/(0.659+2.364+3.217+3.566+1.379+0.938+1.876+0.769+0.367),$$ $$1/\left(0.449+1.825+2.633+2.984+1.058+0.708+1.351+0.548+0.280\right),1/(0.331+1.361+2.000+2.333+0.819+0.533+0.956+0.404+0.230))$$ $$=(\mathrm{0.022,0.038,0.073})$$

Similarly, the remaining ${\stackrel{\sim }{w}}_{i}$ values were calculated, there are:

$${\stackrel{\sim }{w}}_{2}=$$(0.090,0.154,0.264), $${\stackrel{\sim }{w}}_{3}=$$(0.132,0.222,0.359), $${\stackrel{\sim }{w}}_{4}=$$(0.154,0.252,0.398), $${\stackrel{\sim }{w}}_{5}=$$(0.054,0.089,0.154),$${\stackrel{\sim }{w}}_{6}=$$(0.035,0.060,0.105),$${\stackrel{\sim }{w}}_{7}=$$(0.063,0.114,0.209), $${\stackrel{\sim }{w}}_{8}=$$(0.027,0.046,0.086),$${\stackrel{\sim }{w}}_{9}=$$(0.015,0.024,0.041)

The COA defuzzification method (Eq. 12) was used to calculate BNP weights of criteria. For ${BNP}_{1}$ as example:

$${BNP}_{1}=0.022+\left[\left(0.073-0.022\right)+(0.038-0.022)\right]/3=0.044$$

Similarly, the remaining BNP weights of criteria were calculated. After all BNP scores are calculated, normalization is performed for all BNP values.

In regional studies, although the weight value, such as the parent material and lime content, which have little effect on potential agricultural suitability, is lower than other parameters, the degree of slope and effective soil depth which are factors that improving or replacing in the land is the limiting factor for non-economic and agricultural mechanization activities were determined as the criteria with highest weight values. Also, the higher weighting is recommended for properties that pose a continuous risk (erosion)^4,11,84^. Besides, it is known that parameters, which the presence in the environment is not absolutely necessary for vegetative production however effective on soil quality (e.g. organic matter), should have a moderate weight ratio^67^. Based on these evaluations, which the weight values ​​obtained considering the region ecology, the degree of influence of the criteria for determining potential agricultural land was evaluated in 3 groups as high, moderate and low. It was determined that slope (0.245), depth (0.217) and erosion (0.155) criteria are high and texture (0.118), organic matter (0.091) and bulk density (0.061) moderate weight and pH (0.48), parent material (0.041) and lime content (0.024) are low coefficients. As can be seen in this order, the slope criterion with a weight value of 0.245, has obtained as the criterion with the highest weight. Dengiz and Sarıoğlu (2013)^11^ found similar results in their studies and proposed slope should not exceed 10–12% for cultivation without taking soil erosion measures or taking very few measures and therefore, Verdoodt and Van Ranst (2003)^85^ stated that slope is the most important criterion in land capability classification and agriculture practices in field. Because, slope plays an important role in soil erosion and has performing the activities correctly such as in-field mechanization or field traffic. Depth and erosion come in second and third place among the high classification for the criteria being addressed. These criteria are closely related to the retention of water and plant nutrients in the soil and land capability classification, soil fertility and quality characteristics such as plant root development ^41,86^. Considering the ecological characteristics of the region, the parent material in terms of its contribution to soil formation and some agricultural applications (pH regulators, fertilization, etc.) for eliminating the adverse effects of CaCO_3_ content and soil reaction, led the weight values ​​of these criteria were determined to be low.

At the same time, the fact physical properties that can be improved by the improvement of land conditions and organic matter enhancing applications are able to change land suitability and land quality classification positively is an important factor in weighting. As a matter of fact, it is specified that mechanization is facilitated and germination, plant output and yield values increased by the development of the structure by increasing organic matter in agricultural land^87,88^. Besides, the positive effects of the organic matter on water retention, soil compaction, aeration, and biological activity reduce the limiting effect of bulk density and available water capacity and increase soil quality^68,69^.

### Spatial distribution of criteria and potential land suitability for agricultural usages

Site suitability assessment for agricultural applications includes the assessment of a large amount and variety of internal soil condition (depth, organic matter, texture, soil reaction etc.), and external soil conditions (topography, erosion etc.).

**Figure 4:**
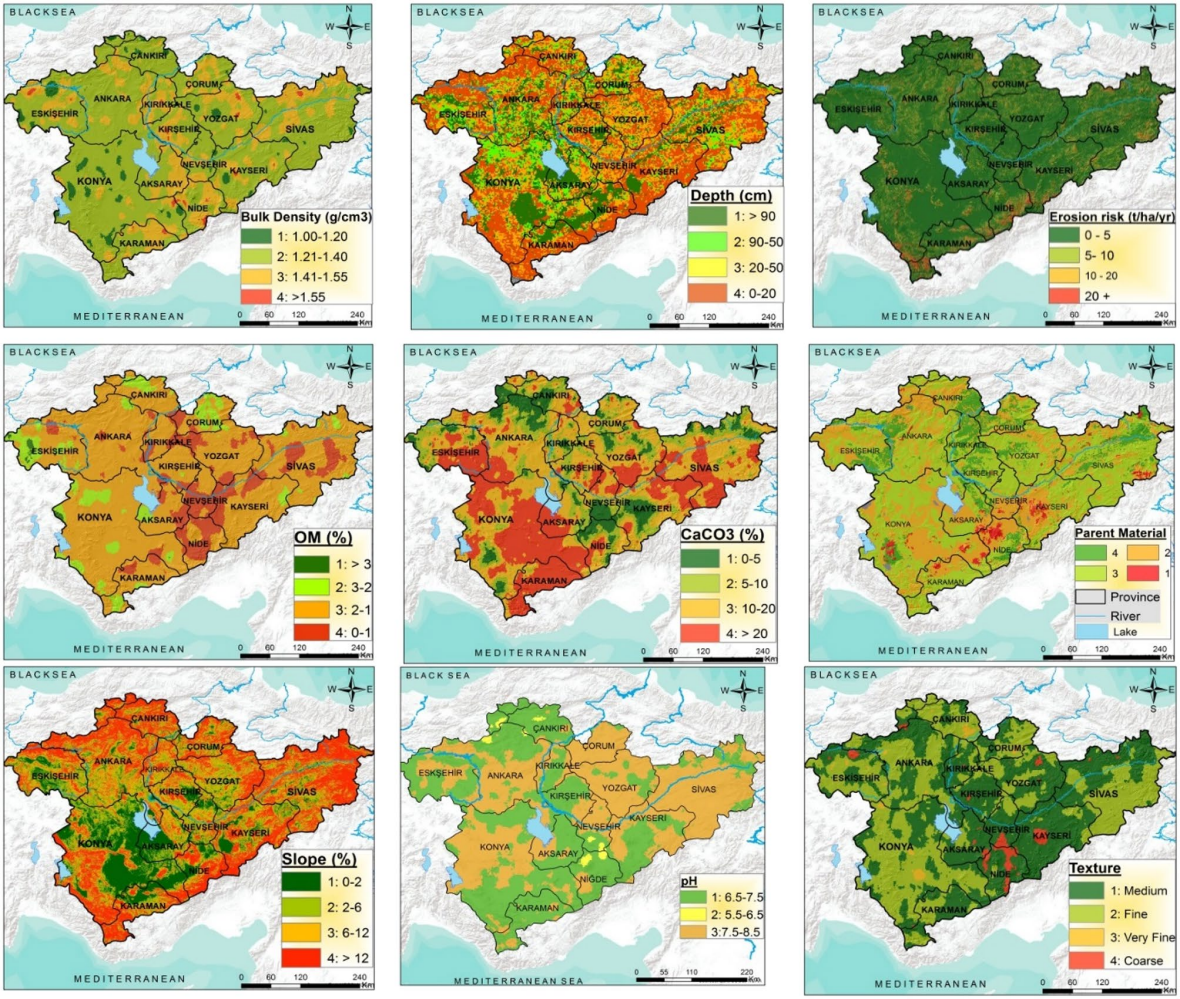
Spatial distribution of some criteria for land suitability for agriculture usages (the maps were created by the authors using the ArcGIS 10.2, http://esri.com).

The areal and proportional distributions of land and soil criteria for identifying the Central Anatolia potential agricultural areas are given in Table 9 and Fig. 4. RUSLE (Revised Universal Soil Loss Equation) model was used to estimate the spatial amount of erosion. It is observed that approximately 7% of Central Anatolia has severe and very severe erosion risk whereas, 83.6% of the region has a slight or low risk of erosion. This situation is parallel with the erosion severity classes of Turkey and according to Turkey's water erosion atlas^89^, the erosion risk of country's surface area consists of 60.28% very low, 19.13% lowt, 7.93% moderate, 5.97% severe and 6.7% very severe. Kosmos et al. (2014)^90^ also used actual soil erosion to define categories of land quality risk, based on the type of environmental sensitivity area (ESA) as an indicator for their empirical approach which was applied in 17 study areas in the Mediterranean region, Eastern Europe, Latin America, Africa and Asia. Moreover, Symeonakis et al. (2014)^91^ estimated the ESAs on the island of Lesvos, Greece through a modified ESA, which included 10 additional indicators related to soil erosion, groundwater quality, demographics and grazing pressure between two years, 1990 and 2000. Results showed that about 85% of the island is fragile or critically sensitive in both periods: 81% in 1990 and 77% in 2000.

**Table 9:** Spatial and proportional distribution of some criteria for land suitability for agriculture usages in Central Anatolia Region.

Criteria	Class	Description	Area	
			ha	%
Erosion (ton/ha/year)	1: 0–5	Very low	16,305,597	83.6
	2: 5–10	Moderate	1,577,389	8.1
	3: 10–20	High	924,183	4.7
	4: 20 +	Very high	694,102	3.6
Depth (cm)	1: 90 +	Deep	3,818,409	19.6
	2: 50–90	Moderate deep	2,637,154	13.5
	3: 20–50	Shallow	5,474,580	28.1
	4: 0–20	Very shallow	7,571,129	38.8
Slope (%)	1: 0–2	Flat	3,845,512	19.7
	2: 2–6	Gently slope	4,360,510	22.4
	3: 6–12	Moderate slope	4,325,500	22.2
	4: 12–20	High slope	6,969,750	35.7
Parent material	1:	–	59,250	0.3
	2:	–	7,267,910	37.3
	3:	–	3,343,580	17.1
	4:	–	8,830,532	45.3
Organic matter (%)	1: > 3	High	95,738	0.5
	2: 3–2	Moderate	3,039,547	15.6
	3: 2–1	Low	11,840,528	60.7
	4: < 1	Very low	4,525,459	23.2
Bulk density (g cm^−3^)	1: 1.00–1.20	Low	806,082	4.1
	2: 1.21–1.40	Moderate	10,760,485	55.2
	3: 1.41–1.55	High	7,832,192	40.2
	4: > 1.55	Very high	102,513	0.5
Texture (%)	1:	Medium	10,300,571	52.8
	2:	Fine	8,607,234	44.1
	3:	Very fine	182,112	0.9
	4:	Coarse	411,355	2.1
pH	1: 6.5–7.5	Slightly acid or alkaline	8,997,319	46.1
	2: 5.5–6.5	Slightly to moderate acid	178,496	0.9
	3: 7.5–8.5	Slightly to moderate alkaline	10,325,457	52.9
	4: < 5.5- > 8.5	Strong acid or alkaline	–	–
Lime content-CaCO_3_ (%)	1: 0–5	Low	3,121,923	16.0
	2: 5–10	Moderate	5,849,441	30.0
	3: 10–20	High	9,401,612	48.2
	4: > 20	Very high	1,128,296	5.8

33.1% of the study area is considered deep, while 38.8% considered very deep for soil depth criterion, which is an important parameter both in plant nutrient and water storage and root development in processed agricultural applications^10,46^. Deep soils are mostly distributed in Konya, Aksaray, in some parts of Ankara, Çorum and Sivas, which are located in the center of Central Anatolia, while shallow soils are widely distributed in the mountainous areas around as well as Karaman, Çankırı, Yozgat and Kayseri provinces. Approximately half of the parent material distribution of the study area (45.3%) consists of acid magmatic, cherty, gneiss, dunes, volcanic ashes, tuff, agglomerate, breccia, evaporites, sand stone, while approximately one third (37.3%) consists of basic-ultra basic magmatic and eruptions, melange, ophiolitic and serpentine, shale, etc. schist, metamorphic rocks, such as phyllite, claystone, and marl. Only very little of the main material (0.3%) consists of young alluvial deposits.

The slope is considered as an important criterion in almost all of the areas for agricultural suitability in evaluation studies. Therefore, the slope degree could be considered a restriction to land capability particularly for irrigated agriculture Sauer et al. (2010)^88^ as it negatively restricts management and machinery applications such as irrigation, tillage and drainage^92^ and determines the type of the irrigation system to be used and the flow rate, hence affecting crop yields and irrigation cost. Slope also affects land productivity as high steep lands suffer from soil loss^41^. According to Table 5, 64.3% of the Central Anatolian lands have a slope less than 12% slope which is the limit value for machinery agriculture, while 35.7% has a high slope. The areas where the slope is flat and moderately sloped are mostly distributed in the central area (Fig. 4). Most of the soils in the area (about 84%) have very low organic matter content, which is usually between 1 and 2%. This situation is also in parallel with Turkey Soil Organic Carbon Study^93^, the area has the highest 67.83 t C ha^−1^ soil organic carbon in the Black Sea Region whereas, it has the second-lowest soil organic carbon stock with 38.5 t C ha^-1^ after Southeast Anatolia Region (29.46 t C ha^−1^) due to low rainfall and vegetation effect.

Approximately 98% of the texture of the Central Anatolian soils are consisting of loam, clay loam, sandy clay loam and clay (< 45% clay content), which are considered medium and fine classes, very few (3.0%) have very fine (> 45% clay content) and coarse (sand, sandy loam and loamy sand) texture. In addition, more than 95% of the soil has medium and high bulk density and they range from 1.21 gr cm^−3^ to 1.55 gr cm^−3^. Central Anatolia Region lands do not contain soils with strong acid pH while more than half of the lands range from slightly to moderate alkali. 16% of the soil has low lime content, while more than half of the lands have high and very high content of lime.

**Figure 5:**
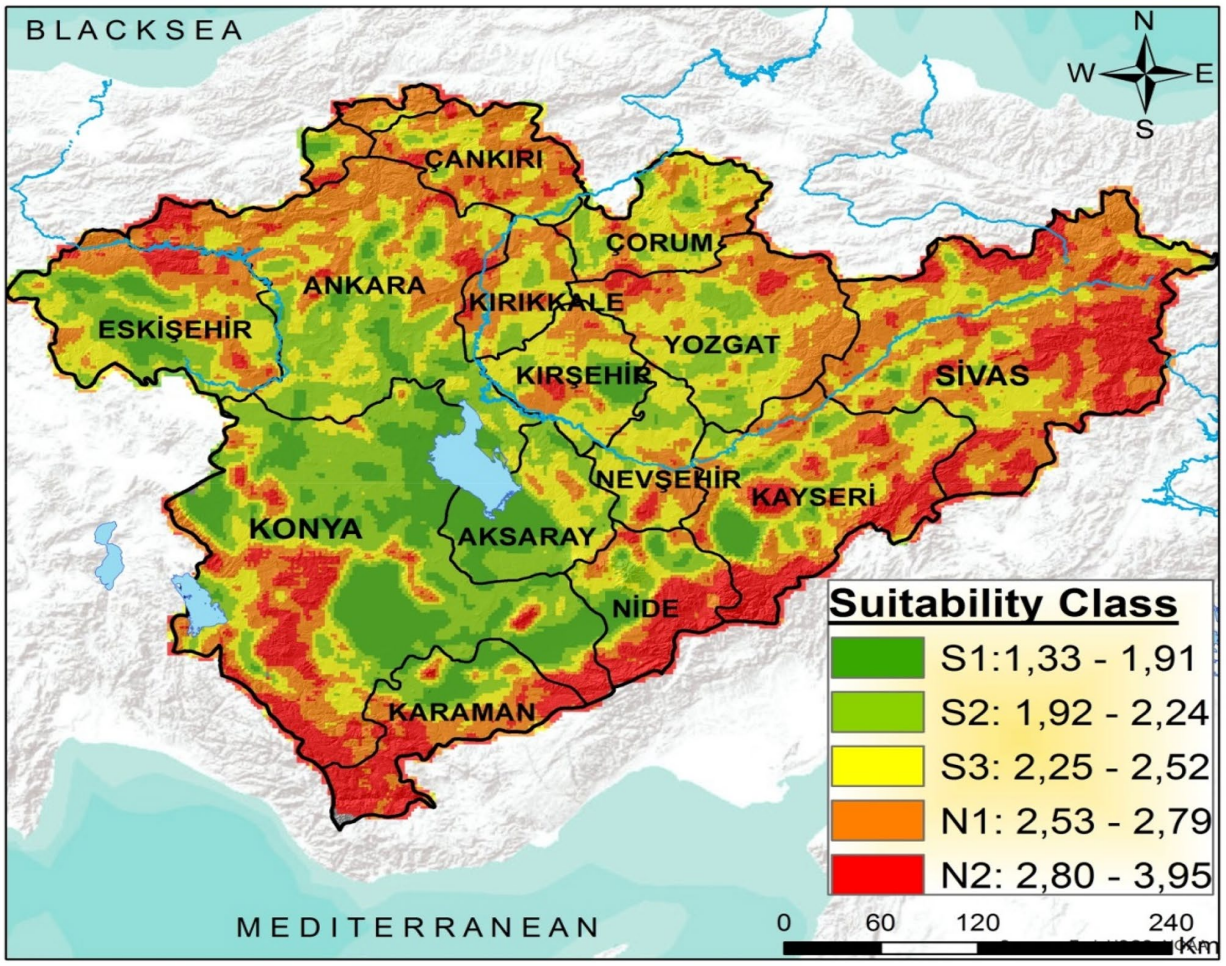
Land suitability classes for agriculture usages of the Central Anatolia Region (the map was created by the authors using the ArcGIS 10.2, http://esri.com).

As a result of the identification of potential agricultural lands of the study area by the FAHP approach, the spatial and proportional distribution of the suitability classifications for each province is given in Table 10 and Fig. 5. Approximately 30.7% (59,922 km^2^) of the total area is determined as being very suitable and suitable for agriculture uses at S1 and S2 levels, whereas 12.6% is not suitable for low-till agricultural activities. Potentially suitable and very suitable areas for agriculture activities are mainly distributed among Konya, Aksaray, Nevşehir, Kayseri, Yozgat, Kırşehir provinces. Areas of the region that are currently or not at all suitable (N1 and N2) for agriculture uses are widely distributed in Sivas, Niğde, Çorum and Kırıkkale provinces and some areas are also identified disconnectedly in the southern part of the study area. The most important factors that restrict agricultural practices in these areas are high slope degree and shallow soil depth. Besides, it was determined that 26.6% of the area is slightly suitable (S3). Among the provinces, Konya has the largest surface area in the Central Anatolia region with an area of ​​38,869.7 km^2^, which corresponds to 19.9% ​​of the total area and 2,354,450 ha area of this area is very suitable (S1) and suitable (S2) areas for agricultural applications as the widest area in the region, and Sivas province has the highest N1 and N2 suitability classes with an area of ​​19,571.6 km^2^ in the Central Anatolia region.

**Table 10:** Land suitability classes for agriculture usages of each province in the Central Anatolia Region.

Provinces		Suitability classes									
		Very high suitable S1		High suitable S2		Marginally suitable S3		Currently non suitable N1		Permanently non suitable N2	
(km^2^)	%	km^2^	%	km^2^	%	km^2^	%	km^2^	%	km^2^	%
Aksaray (7905.1)	4.1	3041.5	1.6	2585.4	1.3	1912.5	1.0	365.8	0.2	0.0	0.0
Ankara (25,142.3)	12.9	1671.0	0.9	7044.5	3.6	7408.3	3.8	7631.0	3.9	1387.5	0.7
Bolu (1585.4)	0.8	119.5	0.1	246.3	0.1	502.8	0.3	554.4	0.3	162.5	0.1
Çankırı (8337.8)	4.3	0.0	0.0	360.0	0.2	2158.5	1.1	5051.0	2.6	768.3	0.4
Çorum (8346.8)	4.3	87.0	0.0	1587.3	0.8	3716.3	1.9	2773.8	1.4	182.5	0.1
Eskişehir (13,709.7)	7.0	1482.8	0.8	3482.8	1.8	3930.8	2.0	3858.2	2.0	955.3	0.5
Kırıkkale (4947.5)	2.6	0.0	0.0	613.5	0.3	1957.5	1.0	2202.5	1.1	174.0	0.1
Kırşehir (6750.7)	3.5	689.5	0.4	1011.5	0.5	3108.3	1.6	1677.0	0.9	264.5	0.1
Karabük (1120.3)	0.6	0.0	0.0	16.5	0.0	170.3	0.1	866.3	0.4	67.3	0.0
Karaman (8619.3)	4.4	1026.0	0.5	1150.8	0.6	983.8	0.5	2379.3	1.2	3079.5	1.6
Kayseri (17,030.2)	8.7	840.8	0.4	2153.8	1.1	4999.8	2.6	5305.3	2.7	3730.7	1.9
Konya (38,869.7)	19.9	11,201.7	5.7	12,342.8	6.3	5313.3	2.7	5947.5	3.0	4064.5	2.1
Nevşehir (5466.9)	2.8	52.3	0.0	1141.0	0.6	2764.5	1.4	1328.8	0.7	180.4	0.1
Niğde (6414.4)	3.3	762.3	0.4	1399.0	0.7	1313.5	0.7	1171.3	0.6	1768.2	0.9
Sivas (26,767.5)	13.9	56.3	0.0	893.8	0.5	6245.0	3.3	12,479.3	6.5	7092.6	3.7
Yozgat (13,999.3)	7.2	15.0	0.0	2847.1	1.5	5360.1	2.6	5130.8	2.6	645.5	0.3
Total area (195,012.7)	100	21,045.7	10.8	38,876.1	19.9	51,845.3	26.6	58,722.3	30.1	24,523.3	12.6

The land capability classification (LCC) system is put into classes ranging from best (Class I) to worst (Class VIII) and gives an indication of the inherent capability of the land for general agricultural production^94^. While it can be assessed I, II, III classes of LCC system suitable for agricultural usage, IV class can be considered as slightly suitable for arable land and other classes are not suitable for cultivation area. General Directory of Rural Service^95^. produced LCC maps in regional and national scales in Turkey. According to GDRS’s report, approximately 33.4% of the total area was found as being in three classes for agriculture uses at I, II, and III, whereas 54.2% is not suitable for agricultural applications. Moreover, it was determined that 12.4% of the area is slightly suitable (IV. class). When compared to current results of the study, amount of agricultural suitable lands decreased about 2.7%, whereas slightly suitable area was significantly changed from 12.4% to 26.6%. On the other hand in the current study non-suitable area was determined 11.2% less. It can be said that these differences resulted from actuality and quality of data, sensitive methodological approach and changings of land use managements.

## Conclusion

Identifying the suitability and quality of the lands has great importance for deciding on the use of land according to its potential and protecting natural resources for future generations. In this study, identification of suitable areas for agricultural land by taking soil and land indicators into account at regional scale carried out in the Central Anatolia Region, which covers approximately 25% of Turkey with 78 million ha. In the current study, land suitability for agriculture usages of the Central Anatolia Region was assessed on the basis of a comprehensive set of criteria associated with multi criteria decision management taking into consideration of the FAHP approach. The integration of fuzzy sets with AHP significantly contributed to the elimination of uncertainties in expert opinions. In light of study results, it was seen that one third % of the study area has high and very high suitable, whereas currently and permanently non suitable areas cover about half of the study area (42.7%), suggesting that the areas are highly sensitive to agricultural activities or cultivations. However, when the results are compared with CORINE 2012 land use-land cover, CORINE 2012 classification shows a distribution of approximately 40% as agricultural area, while this study found that approximately 30% of the area is suitable for agricultural activities but it is also found that agricultural activities take place in areas that are not suitable for agriculture or in marginal agricultural areas, which corresponds approximately 12% of the area. Moreover, this study can contribute important approach by applying fuzzy sets with AHP for land suitability for agriculture usage estimation in regional scale.

Identification of suitable areas for agricultural fields is therefore based on the permanent biophysical features of the land and does not take into account the economics of agricultural production, distance from markets, social or political factors. That is why, this methodology should be integrated with thematic and/or detailed additional information such as climate and socio-economic data, local land use processes and/or yield outputs, and demographics to achieve more sensitive approach for determination of potential site suitability lands for agriculture applications. Moreover, the results of the current study can guide the implementation of the strategic objectives of the National Strategy and Action Plan in order to determine agricultural suitable area for sustainable land recourse.

## References

[1] TÜİK. *Plant Production Statistical Data.**Turkish Statistical Institute* http://www.tuik.gov.tr/bitkiselapp/bitkisel.zul (2018).

[2] FAO. *Water Policy and Agriculture*, *FAO Agriculture Series, No. 26*. (Food and Agriculture Organization, Rome, 1993).

[3] Mueller L, Schindler U, Mirschel W, Shepherd TG, Ball BC, Helming K, Rogasik J, Eulenstein F, Wiggering H (2010). Assessing the productivity function of soils. A review. Agron. Sustain. Dev..

[4] Ahmed, G. B., Shariff, A. R. M., Balasundram, S. K. & bin Abdullah, A. F. Agriculture land suitability analysis evaluation based multi criteria and GIS approach. *IOP Conf. Ser. Earth Environ. Sci.* **37**(1), 012044, 10.1088/1755-1315/37/1/012044 (2016).

[5] Xue R, Wang C, Liu M, Zhang D, Li K, Li N (2019). A new method for soil health assessment based on analytic hierarchy process and meta-analysis. Sci. Total Environ..

[6] Shepherd, T. G. *Visual Soil Assessment. Volume 1. Field Guide for Pastoral Grazing and Cropping on Flat to Rolling Country.* (Horizons Regional Council, Palmerston North, 2009).

[7] Ceballos-Silva A, López-Blanco J (2003). Delineation of suitable areas for crops using a multi-criteria evaluation approach and land use/cover mapping: A case study in Central Mexico. Agric. Syst..

[8] Malczewski, J. Ordered weighted averaging with fuzzy quantifiers: GIS-based multicriteria evaluation for land-use suitability analysis. *Int. J. Appl. Earth Obs.* **8**, 270–277, 10.1016/j.jag.2006.01.003 (2006).

[9] Mandere NM, Persson A, Anderberg S, Pilesjö P (2010). Tropical sugar beet land evaluation scheme: Development, validation and application under Kenyan conditions. GeoJournal.

[10] Akıncı H, Özalp AY, Turgut B (2013). Agricultural land use suitability analysis using GIS and AHP technique. Comput. Electron. Agric..

[11] Dengiz O, Sarıoğlu FE (2013). Parametric approach with linear combination technique in land evaluation studies. Tarim Bilim. Derg..

[12] Demirağ Turan İ, Dengiz O, Özkan B (2019). Spatial assessment and mapping of soil quality index for desertification in the semi-arid terrestrial ecosystem using MCDM in interval type-2 fuzzy environment. Comput. Electron. Agric..

[13] Saaty TL (1980). The Analytic Hierarchy Process.

[14] Uçal Sarı, I., Öztayşi, B. & Kahraman, C. Fuzzy analytic hierarchy process using type‐2 fuzzy sets: An application to warehouse location selection. in (eds. Doumpos, M. & Grigoroudis, E.) *Multicriteria Decision Aid and Artificial Intelligence: Links, Theory and Applications*, 285–308, 10.1002/9781118522516.ch12 (2013).

[15] Kiliç M, Kaya İ (2015). Investment project evaluation by a decision making methodology based on type-2 fuzzy sets. Appl. Soft Comput..

[16] Celik E, Akyuz E (2018). An interval type-2 fuzzy AHP and TOPSIS methods for decision-making problems in maritime transportation engineering: The case of ship loader. Ocean Eng..

[17] Kahraman, C., Öztayşi, B., Uçal Sarı, İ. & Turanoğlu, E. Fuzzy analytic hierarchy process with interval type-2 fuzzy sets. *Knowl-Based Syst.* **59**, 48–57, 10.1016/j.knosys.2014.02.001 (2014).

[18] Buckley JJ (1985). Fuzzy hierarchical analysis. Fuzzy Set. Syst..

[19] Jakhar, R., Verma, D., Rathore, A. P. S. & Kumar, D. Prioritization of dimensions of visual merchandising for apparel retailers using FAHP. *Benchmarking 27*, 2759–2784 (2020).

[20] ZhiGang T, DongDong Z, XiaoJie Y, JiaMin W, Yu S (2020). Evaluation of open-pit mine security risk based on FAHP-extenics matter-element model. Geotech. Geol. Eng..

[21] Al Mamun MA, Howladar MF, Sohail MA (2019). Assessment of surface water quality using Fuzzy Analytic Hierarchy Process (FAHP): A case study of Piyain River's sand and gravel quarry mining area in Jaflong, Sylhet. Groundw. Sustain. Dev..

[22] Rajabi F, Jahangiri M, Molaeifar H, Honarbakhsh M, Farhadi P (2018). Occupational stress among nurses and pre-hospital emergency staff: Application of fuzzy analytic hierarchy process (FAHP) method. EXCLI J..

[23] Bejari H, Daya AA, Roudini A (2017). Selection of chromite processing plant site using fuzzy analytic hierarchy process (FAHP). Int. J. Min. Reclam. Environ..

[24] Kirubakaran B, Ilangkumaran M (2016). Selection of optimum maintenance strategy based on FAHP integrated with GRA–TOPSIS. Ann. Oper. Res..

[25] Nezarat H, Sereshki F, Ataei M (2015). Ranking of geological risks in mechanized tunneling by using Fuzzy analytical hierarchy process (FAHP). Tunn. Undergr. Space Technol..

[26] Andrews SS, Karlen DL, Cambardella CA (2004). The soil management assessment framework: A quantitative soil quality evaluation method. Soil Sci. Soc. Am. J..

[27] Asgari, M. S. & Holden, N. M. Indices for quantative evaluation of soil quality under grassland management. *Geoderma.* 230–231, 10.1016/j.geoderma.2014.04.019 (2015).

[28] Imaz MJ, Virto I, Bescansa P, Enrique A, Fernandez-Ugalde O, Karlen DL (2010). Soil quality indicator response to tillage and residue management on semi-arid Mediterranean cropland. Soil Tillage Res..

[29] Zhang J, Su Y, Wu J, Liang H (2015). GIS based land suitability assessment for tobacco production using AHP and fuzzy set in Shandong Province of China. Comput. Electron. Agric..

[30] Bydekerke L, Van Ranst E, Vanmechelen L, Groenemans R (1998). Land suitability assessment for cherimoya in southern Ecuador using expert knowledge and GIS. Agric. Ecosyst. Environ..

[31] Store R, Kangas J (2001). Integrating spatial multi-criteria evaluation and expert knowledge for GIS-based habitat suitability modelling. Landsc. Urban Plan..

[32] Doran, J. W. & Parkin, T. B. Quantitative indicators of soil quality: A minimum data set. in *Methods for Assessing Soil Quality* (eds. Doran, J. W., Jones, A. J.) Vol. 49, 25–37 (SSSA Special Publications, 1996).

[33] Köksal Taksoy F (2016). Petrogenesis of the Ekecikdağ igneous association (Central Anatolia): Mineral chemistry perspective. Bull. Earth Sci. Appl. Res. Centre Hacettepe Univ..

[34] Öner N, Erşahin S, Ayar S, Özel HB (2016). İç Anadolu’da Yarıkurak Alanların Rehabilitasyonu. Anatolian J. For. Res..

[35] Atalay, İ. Türkiye Topraklarının Oluşumu ve Kullanımı in *Toprak Amenajmanı* (eds. Erşahin, S., Öztaş, T., Namlı, A., Karahan, G.) (Ilksan Matbaası Ltd., Ankara, 2015).

[36] ÇEM. *Kurak ve Yarıkurak Alanlarda Ağaçlandırma ve Rehabilitasyon Rehberi.* T.C. Orman ve Su İşleri Bakanlığı Çölleşme ve Erozyonla Mücadele Genel Müdürlüğü Erozyon Kontrolü Daire Başkanlığı Yayınları, 190. Ankara (2013).

[37] Soil Survey Staff. *Procedures for Collecting Soil Samples and Methods of Analysis for Soil Survey*. (Soil Survey Invest. Rep. I. U.S. Gov. Print. Office, Washington D.C., 1992).

[38] Nelson, D. W. & Sommers, L. E. Total carbon, organic carbon, organic matter. in *Methods of Soil Analysis Part 2. Chemical and Microbiological Properties*, 2nd Edn (ed. Page, A. L.) 539–579 (American Society of Agronomy Inc. Madison, 1982).

[39] Karlen DL, Stott DE, Cambardella CA, Kremer RJ, King KW, McCarty GW (2014). Surface soil quality in five midwestern cropland conservation effects assessment project watersheds. J. Soil. Water Conserv..

[40] De la Rosa, D. & Van Diepen, C. A. Qualitative and quantitative land evaluations. in *Land Use, Land Cover and Soil Sciences,**Vol. II: Land Evaluation* (ed. Verheye, W. H.) 59–77 (Unesco-EOLSS, 1981).

[41] Dengiz O (2007). Assessment of soil productivity and erosion status for the Ankara-Sogulca catchment using GIS. Int. J. Soil Sci..

[42] Hazelton P, Murphy B (2007). Interpreting Soil Test Results: What Do All the Numbers Mean.

[43] Iojă CI, Niţă MR, Vânău GO, Onose DA, Gavrilidis AA (2014). Using multi-criteria analysis for the identification of spatial land-use conflicts in the Bucharest Metropolitan Area. Ecol. Indic..

[44] Mustafa SMT, Vanuytrecht E, Huysmans M (2017). Combined deficit irrigation and soil fertility management on different soil textures to improve wheat yield in drought-prone Bangladesh. Agric. Water Manag..

[45] Demirağ Turan, İ. & Dengiz, O. Çok kriterli değerlendirme ile Ankara Güvenç havzası’nda erozyon risk tahminlenmesi. *Tarim Bilim. Derg.* **23**(3), 285–297, 10.15832/ankutbd.447600 (2017).

[46] Aldababseh A, Temimi M, Maghelal P, Branch O, Wulfmeyer V (2018). Multi-criteria evaluation of irrigated agriculture suitability to achieve food security in an arid environment. Sustainability..

[47] Chen YD, Wang HY, Zhou JM, Xing L, Zhu BS, Zha YC, Chen XQ (2013). Minimum data set for assessing soil quality in farmland of Northeast China. Pedosphere..

[48] Linlin J, Guangming H, Lan Y, Liu S, Gao J, Yang X (2017). Corn cob biochar increasing soil culturable bacterial abundance without enhancing their capacities in utilizing carbon source in Biology Eco-plates. J. Integr. Agric..

[49] Nabiollahi K, Taghizadeh-Mehrjardi R, Kerry R, Moradian S (2017). Assessment of soil quality indices for salt-affected agricultural land in Kurdistan Province, Iran. Ecol. Indic..

[50] Demirag Turan I, Dengiz O, Özkan B (2019). Spatial assessment and mapping of soil quality index for desertification in the semiarid terrestrial ecosystem using MCDM in interval type-2 fuzzy environment. Comput. Electron. Agric..

[51] Eyüpoğlu, F. *Türkiye Topraklarının Verimlilik Durumu.* Toprak ve Gübre Araştırma Enstitüsü Yayınları Genel Yayın No: 220 Teknik Yayın No: T-67, Ankara (1999).

[52] Bajracharya RM, Sitaula BK, Sharma S (2013). Seasonal dynamics, slope aspect and land use effects on soil mesofauna density in the mid-hills of Nepal AU-Begum, Farida. Int. J. Biodivers. Sci. Ecosyst. Serv. Manag..

[53] Sauer T, Havlík P, Schneider UA, Schmid E, Kindermann G, Obersteiner M (2010). Agriculture and resource availability in a changing world: The role of irrigation. Water Resour. Res..

[54] Elsheikh RFA, Abdalla R (2016). Physical land suitability assessment based on FAO framework. IOSR J. Eng..

[55] Sarkar A, Ghosh A, Banik P (2014). Multi-criteria land evaluation for suitability analysis of wheat: A case study of a watershed in eastern plateau region, India. Geo. Spat. Inf. Sci..

[56] Bandyopadhyay S, Jaiswal RK, Hegde VS, Jayaraman V (2009). Assessment of land suitability potentials for agriculture using a remote sensing and GIS based approach. Int. J. Remote Sens..

[57] FAO. A framework for land evaluation. in *FAO Soils Bulletin* Vol. 32. (Food and Agriculture Organization, Rome, 1976).

[58] Feizizadeh B, Blaschke T (2012). Land suitability analysis for Tabriz County, Iran: A multi criteria evaluation approach using GIS. J. Environ. Plan. Manag..

[59] Bera S, Ahmad M, Suman S (2017). Land suitability analysis for agricultural crop using remote sensing and GIS—A case study of Purulia District. Int. J. Sci. Res. Dev..

[60] Pramanik MK (2016). Site suitability analysis for agricultural land use of Darjeeling district using AHP and GIS techniques. Model. Earth Syst. Environ..

[61] Dengiz, O., Turan Demirağ, İ. & Özkan, B. Erzurum ili temel coğrafi özellikleri ve potansiyel işlemeli tarım alanı varlığı. *Atatürk Üniv. Ziraat Fak. Derg*. **50**(2), 136–152, 10.17097/ataunizfd.485163 (2019).

[62] Ashraf MA, Martin S (2002). Identifying critical limits for soil quality indicators in agro-ecosystems. Agric. Ecosyst. Environ..

[63] Şeker C, Işıldar A (2000). Tarla trafiğinin toprak profilindeki gözenekliliğe ve sıkışmaya etkisi. Turk. J. Agric. For..

[64] Pagliai M, Vignozzi N, Pellegrini S (2004). Soil structure and the effect of management practices. Soil Tillage Res..

[65] Gezgin S, Hamurcu M (2006). Bitki beslemede besin elementleri arasındaki etkileşimin önemi ve bor ile diğer besin elementleri arasındaki etkileşimler. Selcuk J. Agr. Food Sci..

[66] Baridón JE, Casas RR (2014). Quality indicators in subtropical soils of Formosa, Argentina: Changes for agriculturization process. Int. Soil Water Conserv. Res..

[67] Riley H, Pommeresche R, Eltun R, Hansen S, Korsaeth A (2008). Soil Structure, organic matter and earthworm activity in a comparison of cropping systems with contrasting tillage, rotations, fertilizer levels and manure use agriculture. Agric. Ecosyst. Environ..

[68] Kurzatkowski D, Martius C, Höfer H, Garcia M, Förster B, Beck L, Vlek P (2004). Litter decomposition, microbial biomass and activity of soil organisms in three agroforestry sites in Central Amazonia. Nutr. Cycl. Agroecosyst..

[69] Guo, L. J., Zhang, Z. S., Wang, D. D., Li, C. F., Cao & C. G. Effects of Short-term conservation management practices on soil organic carbon fractions and microbial community composition under a rice-wheat rotation system. *Biol. Fertil. Soils*. **51**, 65–75 (2015).

[70] Karaca S, Dengiz O, Demirağ Turan İ, Özkan B, Dedeoğlu M, Gülser F, Sargin B, Demirkaya S, Ay A (2021). An assessment of pasture soils quality based on multi-indicator weighting approaches in semi-arid ecosystem. Ecol. Indic..

[71] Malczewski J, Rinner C (2015). Multicriteria Decision Analysis in Geo-Graphic Information Science.

[72] El Alfy Z, Elhadary R, Elashry A (2010). Integrating GIS and MCDM to deal with landfill site selection. Int. J. Eng. Technol..

[73] Jenks, G. F. The data model concept in statistical mapping. in *International Yearbook of Cartography* (ed. Frenzel, K.) Vol. 7, 186–190 (1967).

[74] Zadeh LA (1965). Fuzzy sets. Inf. Control..

[75] Van Laarhoven PJ, Pedrycz W (1983). A fuzzy extension of Saaty's priority theory. Fuzzy Sets Syst..

[76] Hsieh TY, Lu ST, Tzeng GH (2004). Fuzzy MCDM approach for planning and design tenders selection in public office buildings. Int. J. Proj. Manag..

[77] Chang DY (1996). Applications of the extent analysis method on fuzzy AHP. Eur. J. Oper. Res..

[78] Cheng CH (1997). Evaluating naval tactical missile systems by fuzzy AHP based on the grade value of membership function. Eur. J. Oper. Res..

[79] Deng H (1999). Multicriteria analysis with fuzzy pairwise comparison. Int. J. Approx. Reason..

[80] Gumus AT (2009). Evaluation of hazardous waste transportation firms by using a two step fuzzy-AHP and TOPSIS methodology. Expert Syst. Appl..

[81] Sun CC (2010). A performance evaluation model by integrating fuzzy AHP and fuzzy TOPSIS methods. Expert Syst. Appl..

[82] Zhou H, Chen Y, Li W (2010). Soil properties and their spatial pattern in an oasis on the lower reaches of the Tarim River, northwest China. Agric. Water Manag..

[83] Wilding, L. P., Bouma, J. & Goss, D. W. Impact of spatial variability on interpretative modelling. in *Quantitative Modelling of Soil Forming Processes* (eds. Bryant, R. B. & Arnold, R. W.) 61–75 (SSSA, Madison, 1994).

[84] Patrono, A. Multi-criteria analysis and geographic information systems: Analysis of natural areas and ecological distributions. in *Multicriteria Analysis for Land-Use Management* (eds. Beinat, E. & Nijkamp, P.) Vol. 9, 271–292 (Springer, Dordrecht, 1998).

[85] Verdoodt, A. & Van Ranst, E. *A Large-Scale Land Suitability Classification for Rwanda*. 175 (Ghent University, Laboratory of Soil Science, 2003).

[86] Kunda JJ, Nneoma CA, Jajere AA (2013). Land suitability analysis for agricultural planning using GIS and multi criteria decision analysis approach in Greater Karu Urban Area, Nasarawa State, Niger. Afr. J. Agric. Sci. Technol..

[87] Miller, F. & Guthrie, R.L. Classification and distribution of soils containing rock fragments in the United States. in *Erosion and Productivity of Soils Containing Rock Fragments* (eds. Nichols, J. D., Brown, P. L., Grant, W. J.) 1–6 (SSSA, 1984).

[88] Sauer T, Havlík P, Schneider UA, Schmid E, Kindermann G, Obersteiner M (2010). Agriculture and resource availability in a changing world: The role of irrigation. Water Resour. Res..

[89] ÇEM. *Türkiye Su Erozyonu Atlası* 132 (Çölleşme ve Erozyonla Mücadele Genel Müdürlüğü, Ankara, 2018).

[90] Kosmas C (2014). Evaluation and selection of indicators for land degradation and desertification monitoring: Methodological approach. Environ. Manag..

[91] Symeonakis E, Karathanasi N, Koukoulas S, Panagopoulos G (2014). Monitoring sensitivity to land degradation and desertification with the environmentally sensitive area index: the case of Lesvos Island. Land Degrad. Dev..

[92] Lal, R. Soil degradative effects of slope length and tillage methods on alfisols in western Nigeria. I. Runoff, erosion and crop response. *Land Degrad. Dev*. **8**, 201–219, 10.1002/(SICI)1099-145X(199709)8:33.0.CO;2-P (1997).

[93] ÇEM. *Toprak Organik Karbon Projesi*. 36 (Çölleşme ve Erozyonla Mücadele Genel Müdürlüğü, Ankara, 2018).

[94] Erkoçak, A., Dengiz, O. & Kılıç, Ş. Land use capability class data with land forms using GIS case study, Samsun-Bafra District. *Anadolu J. Agric. Sci.* **25**(S-2), 102–107 (2010).

[95] GDRS. General directory of rural service. in *Soil Map Reports at the Scale of 1:100.000, Turkey* (1971).

